# The impact of family stress and resilience on child development: a scoping review

**DOI:** 10.47626/2237-6089-2022-0556

**Published:** 2025-03-27

**Authors:** Marília M. Mendes-Sousa, Marina B. Perrone, Rafael B. de Melo, Marcos V. V. Ribeiro, Qiong Chao, Carolina Torres, Zila M. Sanchez, Pamela J. Surkan, Silvia S. Martins, Thiago M. Fidalgo, Sheila C. Caetano

**Affiliations:** 1 Universidade Federal de São Paulo Escola Paulista de Medicina Departamento de Psiquiatria São Paulo SP Brazil Departamento de Psiquiatria, Escola Paulista de Medicina, Universidade Federal de São Paulo (UNIFESP), São Paulo, SP, Brazil.; 2 Johns Hopkins Bloomberg School of Public Health Department of International Health Baltimore MD USA Department of International Health, Johns Hopkins Bloomberg School of Public Health, Baltimore, MD, USA.; 3 Columbia University Mailman School of Public Health Department of Population and Family Health New York NY USA Department of Population and Family Health, Mailman School of Public Health, Columbia University, New York, NY, USA.; 4 UNIFESP Departamento de Medicina Preventiva São Paulo SP Brazil Departamento de Medicina Preventiva, UNIFESP, São Paulo, SP, Brazil.; 5 Columbia University Mailman School of Public Health Department of Epidemiology New York NY USA Department of Epidemiology, Mailman School of Public Health, Columbia University, New York, NY, USA.

**Keywords:** Family stress, resilience, child development

## Abstract

**Objective:**

Children grow up spending most of their time within the family social environment, where they can experience stressful situations such as marital conflict, a non-cohesive environment, parental alcohol use disorder, parental depression, and other parental mental health issues. All these factors are associated with children's developmental delays. We aimed to conduct a scoping review on associations between family stress and family resilience and child development delays to examine familial conditions associated with child development.

**Methods:**

We conducted a scoping review of observational studies published from January 2000 to July 2023 and indexed in the MEDLINE and LILACS databases. We included observational studies that assessed history of exposure to violence and behavioral or emotional symptoms or mental health problems among children aged 4-12. Data were independently extracted using a structured form.

**Results:**

Database searches identified 12,990 unique records. A total of 43 articles were included in the review. Three main findings emerged: (1) parental mental health problems, especially depressive symptoms in mothers, were associated with child developmental delays and mental health problems; (2) better parenting practices and a cohesive home environment were positively associated with child development; and (3) vulnerable social environments (e.g., poverty and housing insecurity) may be linked to child mental health problems.

**Conclusion:**

The studies reviewed show that promoting better family dynamics and increasing family cohesion, as well as improving parenting abilities, are beneficial to children's socioemotional development and prevention of child mental health problems. Moreover, increasing family and children's resilience improves the quality of life within family units.

## Introduction

Negative experiences and adverse exposures in early childhood increase the risk of poor social, cognitive, and health outcomes.^1,2^ These outcomes could lead to further problems in competence, autonomy, and independence later in life.^3^ Children spend most of their time within the social environment of their families, where they may be exposed to stressful situations such as marital conflict, harsh parenting, non-cohesive environments, and parental mental health disorders, such as depression and alcohol use disorder.^4–6^ All these factors are associated with developmental delays in children.^7,8^

Protective factors for children's socioemotional development have also been studied.^1,9,10^ Resilience has been defined as successful adaptation or functioning in the context of adversity. It occurs in an individual, familial, or community context, and it is associated with social capital and the social environment.^11,12^ Well-adapted parent-child relationships (e.g., affective and supportive) and parental social support can mitigate some problems in developing children's mental health, minimizing the effects of exposure to violence.^13^

Most studies on child development have evaluated the roles of family stress and child resilience separately in small clinical samples.^14–16^ This study aimed to (i) conduct a scoping review on how factors of stress and resilience related to the family environment are associated with socioemotional child development delays and emotional and behavioral problems, and (ii) examine family conditions, such as marital conflicts and parental support, associated with child development and child mental health problems.

## Methods

We conducted a scoping review of studies published from January 1, 2000, to July 31, 2023, indexed in the MEDLINE and LILACS databases. The following inclusion criteria were adopted: studies that (i) included children aged 4-12 years (population); (ii) with adverse family emotional conditions (exposure); and (iii) evaluated the impact of these conditions on children's socioemotional child development, including child behaviors, emotions, and mental health (outcome). Only quantitative studies written in English or Portuguese were included. Exclusion criteria were: (i) study samples with clinical medical conditions, including specific development disorders such as autism spectrum disorder (ASD) or attention-deficit/hyperactivity disorder (ADHD) or learning disorders; (ii) samples studied in non-familial environments such as nurseries; (iii) samples in schools reporting teachers’ perceptions or only academic outcomes; or (iv) studies with fewer than 100 participants, in order to include studies with higher chances of reporting robust findings that studies with smaller sample sizes could not identify.

Our search strategy was "(*family* OR *parent*) AND (*resilience* OR *stress*) AND (*child development*)" in order to capture all related observational studies. In order to assess the grey literature, the references of included articles were also reviewed for additional articles. Additionally, other relevant articles were identified by contacting experts in the field who suggested additional articles to be screened for eligibility. No reference manager was used, and the first authors of the paper were responsible for summarizing all information reviewed, with the supervision of both senior authors.

**Figure 1:**
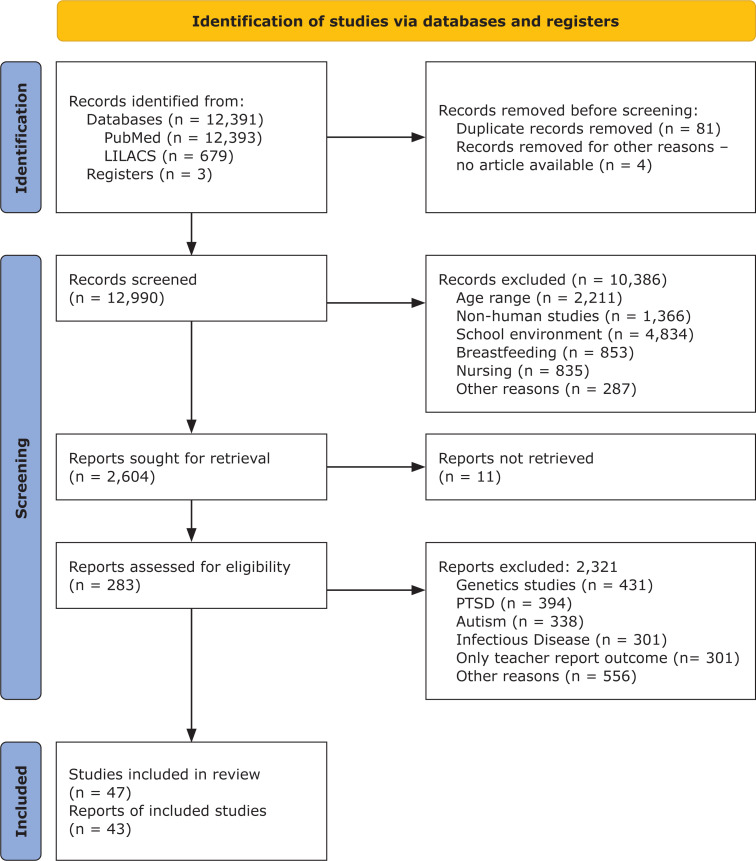
Preferred Reporting Items for Systematic reviews and Meta-Analyses (PRISMA) Flow Diagram of the scoping review of the Impact of Family Stress and Resilience on Child Development. PTSD = post-traumatic stress disorder. From Page et al.^17^

We present the Preferred Reporting Items for Systematic reviews and Meta-Analyses (PRISMA) flow diagram for our review in Figure 1. Database searches identified 13,075 records. We removed 85 duplicate records, leaving 12,990 articles to be screened for eligibility criteria. The main reason for exclusion during the title and abstract screening, full-text screening, evaluation of relevance, and data extraction stages was that the papers were not directly related to our aims. They discussed breastfeeding, school environment, nurseries, solely neurobiological studies, clinical medical conditions (e.g., infectious diseases), ASD, and ADHD. The remaining 43 articles were included in the review.

The articles were independently evaluated by the four authors (MMMS, MBP, RM, QC) responsible for data extraction using a structured form. The following information was collected: (i) author and year of the study; (ii) country where the study was conducted; (iii) sample size; (iv) detailed sample characteristics; (v) age range of the sample; (vi) analysis conducted; (vii) instruments used; (viii) outcome measures; (ix) major findings; and (x) study limitations. Discrepancies were resolved by consensus or by consulting the senior authors (TMF and SCC) when necessary. Information was then analyzed qualitatively. No bias or quality rating scales were used.

This review was registered at PROSPERO under registration number CRD42017068397 and PRISMA guidelines were followed.

## Results

### Study sample

Slightly more than half (53.47%) of the studies were conducted in the United States. Other studies were from the United Kingdom (6.97%), China (6.97%), Israel (4.65%), Canada (4.65%), Germany (4.65%), Thailand (2.33%), the Netherlands (2.33%), Finland (2.33%), Belgium (2.33%), Australia (2.33%), Peru (2.33%), Guyana (2.33%), and Portugal (2.33%). Study designs were cross-sectional (n = 13, 30.22%) or longitudinal (n = 30, 69.78%). Sample sizes ranged from 102 to 21,527. Child emotional and behavior problems (62.79%) and child development delays (18,61%) were the primary outcomes in most studies.

### Cross-sectional studies

Details of the cross-sectional studies are presented in Table 1. The 13 cross-sectional studies were published between 2004 and 2023 and the number of participants ranged from 116 to 21,527.^18,19^ Risks for developmental delays were assessed in three of these studies, but no standardized instruments were used.^20–22^ Positive parenting practices such as family meals and singing or reading to children were associated with better child development.^20,23–25^ Adverse family environments that included child maltreatment and neglect were associated with social and emotional developmental delays.^23,26^ Parents’ excessive use of mobile devices can impair their ability to respond to children's emotional cues and regulate their behavior.^27^

**Table 1:** Cross-sectional studies - Family vs. child development (n = 13)

Authors	Country	Sample size	Sample details	Age range in years	Analysis	Instruments	Outcome measure	Major findings	Study limitations
Vreeland et al.^18^	USA	117	Mothers and their children from Southeast USA community	9-15	MLR	CBCL + YSR RSQ-FS SCID BDI	Adolescent INT and EXT Adolescent coping and stress reactivity Maternal depressive symptoms and diagnosis	Youth's coping and stress reactivity moderated association between current maternal depressive symptoms and youth symptoms Maternal depressive symptoms: associated with youth's INT and EXT when youth used low (vs. high) levels of control coping; associated with youth symptoms for those with ↑ stress reactivity and involuntary disengagement.	Sample limited regarding range of maternal education and income (i.e., primarily middle and upper SES) No information on severity and chronicity of maternal history of depression and other types of psychopathology
Cprek et al.^20^	USA	21,527	NSCH 2011/2012	1-5	MLR	PEDS	Parent-reported risk of developmental, behavioral, or social delays	Positive parenting practices correlated with child's risk of developmental, social, or behavioral delays, in a dose-response relationship	Recall and social desirability biases: ↑ positive parenting practices Scores calculated: parent concerns of delay
Price et al.^26^	USA	177	Home environments of maltreated and non-maltreated children	4-6	Hierarchical regression analyses	CBCL Post-Visit Inventory	Child EXT	Family environments of physically abused children: ↑ negative social interactions Home environments of neglected children: ↓ organized and clean Predictive of EXT: physical abuse, mother's negative behavior toward the child, aggression between siblings, home not organized or clean	No information on maltreatment status at evaluation time
Sharabi et al.^23^	Israel	287	Grades 5-6 from five elementary schools	10-11	MANOVA Hierarchical regression analyses	LS-H CSOC FACES III Hope Scale Effort Scale	Loneliness Sense of coherence	Children in cohesive families: ↓ loneliness and ↑ personal strengths Children in rigid and non-cohesive family: ↑ loneliness and ↓ coherence	Evaluation of children's perceptions of the family dimensions, and not their parents’ perception
Nanthamongkolchai et al.^21^	Thailand	320	Child caregivers for the past 6 months	6-12	MLR	TONI-3 test Child Rearing Scale	Development of children	Children reared by a grandparent: 2 × greater chance of having delayed development vs. those reared by parent	Non standard instruments
To et al.^22^	Canada	4,987	Canadian NLSCY	1-5	MLR	PPVT-R CES-D	PDA	↑ risk of PDA: 1-year-old with ↓ birthweight, being male, having immigrant mother ↑ risk of PDA: 4-5 years old with immigrant mother and mother with low educational attainment Predictor of PDA across all ages: ↓ income	PDA tests used verbal skills: mothers who were immigrants may be disadvantaged
Zhang et al.^28^	China	777	Two-parent families and their school-age children	10-15	SEM	Net family income *per capita* Family debt-to-asset ratio Economic pressure K6 MPMS NIPI Child self-concept	Economic pressure Parental emotional distress Marital satisfaction Nurturant-involved parenting Child emotional distress Child self-concept	Economic pressure and parental emotional distress: role in the connection between family economic difficulties and child emotional distress Parents’ emotional distress: mediator between economic adversities and children's emotional well-being (particularly in rural families)	Economic pressure: assessed by single question Important constructs not included (e.g., marital conflict) Constructs of marital satisfaction and emotional distress: responses from only one parent Limitations in generalizing to other cultures and contexts
Miller-Graff et al.^24^	Peru	385	Caregiver-child dyads	4-17	MLiR	SDQ DQ JVQ PSQ FACES-IV PBS RRC-ARM PHQ-9	Child adjustment difficulties Children's prosocial skills	↑ child victimization: ↑ child adjustment problems ↑ caregiver depression and harsh parenting practices: ↑ child adjustment problems ↑ caregiver depression: ↑ child prosocial skills ↑ positive parenting, caregiver resilience, and family cohesion: ↑ child prosocial skills: ↑ environmental promotive factors Family cohesion predicted child prosocial behaviors	Limitations of single informants Reporting bias No information on caregiver physical abuse Diverse age range of children
Roopnarine et al.^29^	Guyana	196	Mother-preschool child dyads	3-5	PA (mediating roles)	CBCL CES-D RCTS Physical Assault Subscale Constructive conflict behaviors between couples Partner social support	Constructive conflict behaviors Maternal depressive symptoms Intimate partner violence Child INT and EXT	Constructive conflict behaviors partially mediated the link between maternal depressive symptoms and EXT. Maternal depressive symptoms and physical intimate partner violence: related to EXT. Families with ↑ partner social support: constructive conflict behaviors appeared to be more effective in ↓ EXT.	Data collection during COVID-19 pandemic: could have ↑ depressive symptoms and physical intimate partner violence Reports of children's INT and EXT not obtained from teachers No data on social support offered by friends, relatives, and community.
Nikstat et al.^25^	Germany	2,089	Twin pairs and families from two twin birth cohorts (ages 11 and 17) in wave 1 of the German TwinLife study of social inequalities	1,043 11-year-old twin pairs 1,046 17-year-old twin pairs	CFA	SDQ 16 indicators of family environment (e.g., SES, positive parenting) ZQYT SRZQ	Vantage sensitivity model Positive parental moderators Child INT	Indicators of family environment derive four dimensions: positive parenting, negative parenting, lack of parental resources, and SES Mechanism of gene-environment interaction with increasing nonshared environmental variance for ↑ adverse and ↓ propitious family conditions In preadolescence, parenting behavior: greater moderating influence on INT vs. family conditions (e.g., SES)	Nonshared environmental variance component may contain measurement error Nonlinear G × E models results suggest that family environments might not moderate genetic and environmental variance linearly Factor scores for positive and negative parenting included individual data for both twins: possibility of inflated false positives INT: self-reports
Cui et al.^30^	China	150	Parent-child dyads	6-12	PA	PSI-SF DERS CCNES CPPSC-SF	Associations between parents’ perception of child difficultness/emotion dysregulation and parents’ supportive/unsupportive reactions to children's negative emotions Relationships between parents’ perception of child difficultness, parents’ emotion dysregulation, and parents’ psychological control behavior concerning children's thoughts and guilt induction	Perceived child difficultness: ↓ parental supportive reactions Parental emotion dysregulation: ↑ parental unsupportive reactions Interaction between perceived child difficultness and parental emotion dysregulation predict psychological control Parental emotion dysregulation exacerbated effect of perceived child difficultness on parental psychological control	Self-report bias Observed psychological control: ↓ variability and not related to child outcomes Generalization limitations Need for broader measures of child characteristics Role of siblings or other family members in influencing parenting dynamics: not considered
Watson et al.^19^	USA	116	Mothers and their children	9-15	MLR	CBCL + YSR BDI-II RSQ-FS Observational coding system for maternal coping socialization messages	Child INT Association between coping socialization messages and INT Moderation by maternal depressive symptoms and peer stress	Child peer stress: positively correlated with INT Maternal depressive symptoms: positively correlated with INT moderate link between coping messages and self-reported INT At ↑ maternal depressive symptoms: ↓ maternal guidance on primary control and secondary control coping - had children with ↑ INT At ↑ peer stress, mothers who encouraged less secondary control coping had children with ↑ INT	Sample limitations Coding system for analyzing coping messages: requires additional validation No data on how parents influence coping responses from infancy through adulthood
Lv et al.^27^	China	988	Mothers of young children	3-6	PA (mediating and moderating roles)	SDQ PS PAS PSI-SF	Maternal phubbing Mother-child attachment Parenting stress Emotional and behavioral problems in children	Mothers’ phubbing: negatively associated with child emotional and behavioral problems Parents’ phubbing: negatively affected parent-child relations and children's emotions Maternal parenting stress moderated relation between mother-child attachment and children's emotional and behavioral problems Mother-child attachment mediated relation between mother phubbing and child emotional and behavioral problems	Mothers’ self-report data

BDI = Beck Depression Inventory; CBCL = Child Behavioral Checklist; CCNES = Coping with Children's Negative Emotions Scale; CES-D = Center of Epidemiological Studies-Depression Scale; CFA = confirmatory factor analyses; COVID-19 = coronavirus disease 2019; CPPSC-SF = Chinese Parental Psychological Control Scale Short Form; CSOC = Children's Sense of Coherence Scale; DERS = Difficulties in Emotion Regulation Scale; DQ = Demographic Questionnaire; EXT = externalizing problems; FACES III = Family Adaptability and Cohesion Evaluation Scale; FACES-IV = Family Adaptability and Cohesion Scale; G × E = genes × environment; INT = internalizing problems; JVQ = Juvenile Victimization Questionnaire; K6 = Kessler Psychological Distress Scale; LS-H = Loneliness Scale – Hebrew adaptation; MANOVA = multivariate analysis of variance; MLiR = multiple linear regression; MLR = multiple logistic regression; MPMS = maternal and paternal marital satisfaction; NIPI = nurturant-involved parenting inventory; NLSCY = National Longitudinal Survey of Children and Youth; NSCH = National Survey of Children's Health; PA = path analysis; PAS = Parent-Child Attachment Scale; PBS = Parent Behavior Scale; PDA = poor developmental attainment; PEDS = Parents’ Evaluation of Developmental Status Questionnaire; PHQ-9 = Patient Health Questionnaire-9; PPVT-R = Peabody Picture Vocabulary Test-Revised; PS = Phubbing Scale; PSI-SF = Parenting Stress Index-Short Form; PSQ = Prosocial Skills Questionnaire; RCTS = Revised Conflict Tactics Scale; RRC-ARM = Resilience Research Centre-Adult Resilience Measure; RSQ-FS = Responses to Stress Questionnaire-Family Stress version; SCID = Structured Clinical Interview for Diagnostic and Statistical Manual of Mental Disorders (DSM); SDQ = Strengths and Difficulties Questionnaire; SEM = structural equation modeling; SES = socioeconomic status; SRZQ = Self Report Zygosity Questionnaire; TONI-3 = Test of Nonverbal Intelligence; YSR = Youth Self-Report Scale; ZQYT = Zygosity Questionnaire for Young Twins. ↑ = higher or increased; ↓ = lower or decreased.

### Longitudinal studies

Thirty studies had a longitudinal design, all published between 2000 and 2023.^1,2,5-8,10,11-16,31-49^ The duration of follow-up in these studies ranged from 1 to 10 years. The number of waves varied from one to six. The Child Behavioral Checklist (CBCL) (n= 13), Strengths and Difficulties Questionnaire (SDQ) (n= 6), Center of Epidemiological Studies-Depression Scale (CES-D) (n= 5), and Child Depression Inventory (CDI) (n= 5) were the most frequently used instruments.^1,2,5-7,10,11,14,16,31-35,40,42,44-49^

Almost half (43.33%) of these studies reported associations between parents with depression and child internalizing symptoms and behavioral problems.^2,5,6,8,11,12,16,33-36,40,47^ Poor marital relationships negatively affected parent-child interactions and were associated with developmental delays and internalizing or externalizing problems.^1,2,4,5,15,16,36,42,46,49^ Also, problematic drinking has been associated with marital aggression.^8,32^ Keller et al.^8^ found that this combination seems to lead to development of adverse reactions to conflicts and low expectations for their children's future. The authors also emphasized that children tend to develop sad reactions related to their mothers with alcohol-related problems and anger reactions to their fathers.

Violence or punishment practices associated with the family environment have also been studied.^5,11,35,44,45^ Parental negligence and violence were associated with externalizing problems and negative social interactions in their children, and decreased home organization and cleanliness.^2^ Specific to girls, maladaptive marital conflict was associated with internalizing problems. When presented with one or more scenarios depicting interparental conflicts, 88% of the children reported a caregiving response. These caregiving responses were categorized as follows: offering comfort (37%), providing assistance (31%), and mediating the situation (63%).^46^ Nonetheless, adolescents who experienced greater interparental conflict during their childhood exhibited heightened negative emotional reactivity in response to family conflicts.^42^

Dubowitz et al.^11^ found that 48% of their 943-child sample presented adequate levels of resilience. These resilient children had less history of maltreatment, caregivers with fewer depressive symptoms, and fewer unemployed caregivers. Households with a small number of members tended to have more resilient children. Savage-McGlynn et al.^12^ found that a mother's positive perspective toward the parenting role favored resilience to the adverse effects of postnatal depression. They also found that 15-month-old children who were more able to communicate non-verbally had a higher likelihood of being resilient at 11 years of age. In the same direction, positive maternal feelings about parenting and good child non-verbal communication at 15 months increased the likelihood of later adequate resilience levels.

Details of the longitudinal studies are presented in Table 2.

**Table 2:** Longitudinal studies - Family vs. child development (n = 30)

Author	Country	Sample size	Sample details	Age range in years	Analysis	Instrument	Outcome measure	Major findings	Study limitations
Zemp et al.^1^	Germany	809	German Family Panel study (pairfam)	7-16	LCA	SDQ DCI NRI MCQ CRI	Child INT and EXT Interparental interaction	Parents in ↓ positivity and ↑ negativity interaction: ↑ INT Parent-child conflict predicted initial INT and EXT	Couple behaviors: measured by reports of the anchors about their partners’ behavior 2-year time lag between measurement occasions: too long
Ramchandani et al.^2^	UK	13,351	Participants from ALSPAC	3-5	SEM MLR	SDQ EPDS RRPS	Child behavior problems Child INT	Paternal depression: couple conflict and maternal depression, which lead to poor children's outcome Maternal postnatal depression: affects children through direct mother-infant interaction and care	Assessment of antisocial traits: scales not validated Response rates: lower for fathers
Choi and Becher^5^	USA	1,773 nonmarital families	FFCWS		SEM	CBCL Economic hardship CIDI-SF Parenting stress Supportive coparenting CTSPC	Child EXT Harsh parenting Supportive coparenting Parenting stress	↑ supportive coparenting: associated with ↓ child behavioral problems and less harsh parenting Maternal depressive symptoms: indirectly and positively related to harsh parenting practices and child behavior problems, transmitted through supportive coparenting and parenting stress acting as mediator Older maternal age: protective for supportive coparenting	Interviewed one member of the coparenting relation Did not rule out the effects of broader environmental systems (school, nonparent caregivers, health care systems, public assistance, neighborhood characteristics, community resources) Coparenting scale does not capture several domains of coparenting (e.g., exposure to conflict, undermining)
Cummings et al.^6^	USA	235	Community families from South Bend and Rochester and surrounding areas	5-6	SEM	CBCL CES-D NEs-SEFQ-SF SIMS-PR	Child INT Child's emotional insecurity	↑ depressive symptoms in both mother and father ↑ INT positive correlation with child's negative emotional expressiveness and insecurity	Parents’ reports of their children's INT may be biased
Letourneau et al.^7^	Canada	10,033	Canadian NLSCY	2-11	MLR	CES-D	Child development in cognitive and behavior domains	Children of depressed mothers: ↑ risk of low receptive vocabulary, displaying inattention, physical aggression Mothers who experienced depression continuously or when child ≥ 2-3 years: ↑ risk of poor emotional development	Self-report biases
Keller et al.^8^	USA	235	Community sample of families living in midsize towns and their surrounding areas	Children in kindergarten	SEM	PAES CPS-Verbal Aggression and Physical Aggression	Child emotional insecurity	Greater parental problem drinking children's negative emotional reactions to conflict ↑ negative expectations for the future indirectly associated with child reactions to marital aggression Mother problem drinking: ↑ sad reactions Father problem drinking: ↑ anger reactions	Difficult to generalize results Single indicators of each construct
Fletcher et al.^10^	Australia	2,620	LSAC Depression: both parents	4-5	MLR	SDQ Derived Outcome Indices	Child behavior problems Social and emotional development	Early paternal depression: predict poorer child outcomes for ↓ development and well-being score	Biased sample (highly educated subjects, with high income, and high full-time job)
Dubowitz et al.^11^	USA	943	LONGSCAN	4-6	MLR	CBCL BDIST WPPSI-R CES-D	Functioning in behavioral, social, and developmental domains	Resilient: 48% of sample Resilient children: ↓ history of maltreatment, ↓caregiver with depressive symptoms, ↑ smaller households, ↑ employed caregivers Poor outcomes: child maltreatment and caregiver depressive symptoms	Genetics or psychosocial interventions may have influenced outcomes Defined competencies and resilience in varying ways: specific normative threshold or relation to other in sample
Savage-McGlynn et al.^14^	UK	6,500	Pregnant women, living in the former Avon Health Authority (England)	8 months-12 years	SEM	SDQ EPDS MCDI HOME	Normative behavior development	Mother's positive perspective of parenting role: ↑ development of resilience to the adverse effects of postnatal depression 15-month-olds who were more able to non-verbally communicate: ↑ likelihood resilient at 11 years of age Maternal positive feelings about parenting and child non-verbal communication at 15 months: ↑ likelihood of later resilience.	EPDS: not a diagnostic instrument Measures: based on maternal reports from ALSPAC sample Sample not representative of UK population
Brock et al.^15^	USA	102	Two-parent community families from US Midwest	2-10	Bootstrapping sampling Nonparametric resampling	CPS AQS-3 CSI-4 ECBQ	Child INT Maladaptive conflict	Maladaptive marital conflict: ↑ INT for girls Negative emotional aftermath of conflict: ↑ INT for boys and girls	Small sample size Sample was relatively ethnically homogenous and low risk
Hazel et al.^16^	USA	692	3rd, 6th, 9th grades, followed every 3 months for 1 year	7-16	Mixed effect models	CDI NRI ALEQ	Youth depressive symptoms Stressful life events Peer stress	Parent relationship quality moderated relationship of person level fluctuations in peer stressors Association between peer stressors and ↑ in depressive symptoms in youth with ↓ positive parental relation	Relationship quality: measured only at baseline and reported by only one parent Other stressors (e.g., health stressors): not evaluated
Kok et al.^31^	Netherlands	1,800	Generation R Study and NICHD SECCYD	0-17.5	SEM	CBCL Ainsworth scales for sensitivity and cooperation Erickson scales for supportive presence and intrusiveness	Child INT Maternal sensitivity	Maternal sensitivity and INT: associated during preschool years	Sample not representative, with no Hispanic participants Mothers’ characteristics influence validity of child perception
Sandler et al.^32^	USA	162	Mothers divorced no longer than 2 years	9-12	SEM	CBCL + YSR DESC PERI-D CPIC CCSC CDI RCMAS	Child INT Psychological problems	Active coping efforts: ↑ efficacy of coping Coping efficacy mediates active coping efforts and psychological problems of children of divorce mediate active coping and EXT and ↓ INT Indirect effect: active coping → ↓ symptom intercept for child reports	Active coping and efficacy of other ways of coping may overlap and lead to misinterpretation
Chan et al.^33^	USA	171	40% mothers: depression-spectrum disorder during child's lifetime	8-12	MLR	SCID-DSM-IV CDI UCLA-LSIC	Dependent family stress	Baseline level of depressive symptoms predicted generation of dependent family stress 1 year later Chronic strain and family factors influence stress generation process	Follow-up time point: entered the pubertal transition Memory biases
Leinonen et al.^34^	Finland	527	Children's mental disorders in Finland	12	ANOVA	CBCL + YSR GHQ-28 CDI	Child adjustment mental health	Parental mental-health problems compromise parenting abilities and represent a threat to children's adjustment	Small effect sizes Only children in early adolescence Single-parent families not included
Black et al.^35^	USA	194	Preschool children born when their mothers were ≤ 19 years and living with their biological mothers	4-5	MLR	CBCL CES-D Apgar score AAPI BDIST	Child INT and EXT Child development	↑ EXT among the highest risk group: maltreated children and children with mothers with depressive symptoms ↓ EXT: children living with mothers in own house (often also living with father), children who had not been maltreated and who had mothers with ↓depressive symptoms	Specific measure of quality of all relationship among different family members not included
Vakrat et al.^36^	Israel	1,983	Women recruited on the second postbirth day, Jan/02 to Mar/05, completed at least 12 years of education, cohabitating with infant's father	0-12	MANOVA	BDI STAI SCID-DSM-IV CIB	Dyadic interaction Parent sensitivity Parent intrusiveness Child social engagement Triadic family interactions Family cohesion	Maternal depression: ↓ parent sensitivity, ↑ parent intrusiveness, ↓ child social engagement with parent, ↓ family cohesion Parental sensitivity: ↓ parental intrusiveness, ↓ child engagement with mother Maternal sensitivity: associated with father sensitivity and child social engagement with father ↓ Father sensitivity or ↓ involvement with father or ↑ father intrusiveness: maternal depression negatively impacted family cohesion Father intrusiveness: ↓ child involvement with father, ↓ family cohesion ↑ family cohesion: ↑ family rigidity	Unmeasured: psychological, genetic or contextual factors Not observed: longitudinal father-infant interactions in 1st year of life Extreme-case design Not included: anxiety disorders Not tested: other family constellations
Waters et al.^37^	Belgium	157	3-year longitudinal study (4th-, 5th-, 6th-grade) yearly laboratory visits Apr/2013 to Sep/2016	9-13 years in the first wave, 4-wave - each year	SEM	Childhood Attachment Interview ASA (Childhood, Adolescent, Adult) ALEQ	Stability of secure base script knowledge Convergent validity of secure base script	Daily hassles (minor and frequently occurring stressful life events) but not major (more severe and infrequent) predicted change in script knowledge Secure base script knowledge: stable across all four waves	Same measure administered across waves: may have inflated stability coefficients Number of time points collected prevented from exploring more complex developmental dynamics of stability of attachment security
Khafi et al.^38^	USA	193	Community-based child development centers and preschools	Preschool children to first graders	ANOVA	EOI FMSS TOF VBD-WPPSI-III PSI SES SILS	Behavior problems from preschool to first grade	Statements of attitude and self-sacrifice/overprotection in FMSS fulfilling EOI criteria: ↑ EXT Effect of Statements of attitudes on EXT for boys	Limited assessment of INT Sample size: limited to differ ethnicity
Wang et al.^39^	USA	4,898	Children and mothers	3-15	SEM	NCP NCE 12-item scale from CDS Exposure to ACE Adolescents’ Behavior Problems self-report scale Adapted scale of NLSAH Self-report SSRS	Adolescents’ behavior problems Adolescents’ delinquency Adolescents’ social skills	Neighborhood collective efficacy: ↑ adolescents’ delinquency and behavior problems, and ↓ social skills Maternal parenting stress and exposure to ACEs: mediators between neighborhood characteristics and adolescents’ outcomes Parenting stress mediated relations between neighborhood poverty and collective efficacy and adolescents’ outcomes Predictors of adolescents’ behavior problems: maternal parenting stress, exposure to ACEs, neighborhood collective efficacy, and mothers’ cohabitation status. Predictors of adolescents’ delinquency: mothers’ marital status, ACEs, mothers’ age, and children's sex. Predictors of adolescents’ social skills: collective efficacy, maternal education, marital status, and parenting stress.	Self-report bias Single time point outcomes Roles of fathers and other caregivers, peers and school: not considered Final model explained modest portion of the variance in each dependent variable, suggesting that additional factors not considered in the model contribute to adolescent outcomes
Guerrero et al.^40^	USA	3,630	Families at birth and ages 1, 3, 5, and 9	1-9	SEM	CBCL CIDI-SF CFSS Questions about homelessness risk	Child INT and EXT Maternal depression Food insecurity Housing instability	↑ Maternal depression at years 1 and 3: ↑ child problem behaviors at year 9 Food insecurity and housing instability at year 5: partly mediated relation between maternal depression at year 3 and EXT at year 9 Food insecurity at year 5 mediated relation between maternal depression and INT or INT and EXT	Sample attrition Missing measurements Assessment bias Instrument limitations Food insecurity measurement
Mason et al.^41^	USA	313	Preschool-age children and their parents and teachers	3-5	SEM	Working memory, inhibitory control and cognitive flexibility WJ-III Brief Intellectual Assessment Subtests LISRES SWPS EC-HOME Questions of parent substance use Questions of adaptive and maladaptive functioning in middle childhood	Adaptive and maladaptive functioning in middle childhood	↑ socio-familial stress, parent smoking, and male sex: ↑ maladaptive and ↓ adaptive functioning in middle childhood ↑ EC and FCA: ↓ maladaptation and ↑ adaptation in middle childhood Socio-familial stress and parent smoking: ↓ FCA Socio-familial stress factors predicted: FCA more strongly than EC FCA with ↑ parent social stress and financial stress and ↓ household stress Predictors of maladaptive functioning: EC and FCA, along with parent social stress and sex Predictors of adaptive functioning: FCA, parent social stress and sex Mediation for socio-familial stress, sex, and parent smoking on both maladaptation and adaptation through FCA	Limitations in sample characterization Modeling strategy limited inclusion of additional intervening variables due to number of parameter estimates and sample size
Davies et al.^42^	USA	235	Mothers, fathers, and their kindergarten children	Wave 1: average age of 6 years; wave 2: average age of 13 years	SEM	CBCL SDQ IPST SCID FPST SSB-A	Interparental conflict history composite Contemporaneous interparental conflict composite Adolescent emotional reactivity to family conflict composite Youth psychological problems composite Adolescent negative schemas about mild and intense family conflicts	Childhood interparental conflict moderated link between recent interparental conflict and adolescents’ negative emotional reactivity Adolescents with ↑ interparental conflict during childhood: ↑ negative emotional reactivity to family conflict Adolescents exposed to ↑ recent interparental conflict: ↑ negative emotional reactivity irrespective of their interparental conflict history Emotional reactivity to family conflict predicted psychological problems in adolescents Negative schemas activated by mild family conflicts partially mediated association between childhood interparental conflict and negative emotional reactivity to family conflict in adolescence	Limitations in sample characterizations Demographic bias Measurement of schemas at a single time point Effect sizes: modest to moderate Deficit in exploring biological or genetic factors
Ferreira et al.^43^	Portugal	214	Children and their parents and teachers	3-6	SEM	6-items of WFCS PRQ-Preschool Form SSRS STRS	Work-family conflict Maternal relational frustration Child social skills Teacher-child conflict	Mothers: moderate levels of work-family conflict and maternal relational frustration in different time points ↑ maternal relational frustration: ↓ child self-control Child self-control: negatively associated with teacher-child conflict across time points mediate relations between maternal relational frustration and teacher-child conflict	Descriptive nature of data Causal modeling Autoregressive cross-lagged panel models: limitations in disentangling between-person and within-person variations Limitations in modeling strategies Unconsidered factors Shared method bias Generalization limitations
Yoon et al.^44^	USA	771	Adolescents from NSCAW-II	11-17	MLR	CBCL + YSR CDI SSRS LSDQ WJ-III DFSCA VEX-R DPAS Abridged Community Environment Scale	INT and EXT domains Social domain Cognitive domain resilient functioning of adolescents over time	Moving from less to greater resilience: early adolescents: ↑ odds predicted by better parent-child relation quality Remaining in greater resilience: ↑ odds: early adolescents, better parent-child relation quality and neighborhood safety ↓ odds: physical abuse history Moving from greater to less resilience: early adolescents: ↓ odds Affiliation with deviant peers: ↓ odds	Generalization limitations Selection bias Self-reported data No differentiation on specific maltreatment subtypes Resilience measures Limited temporal context
Marçal et al.^45^	USA	2,719	FFCWS (unmarried parent)	Waves at age 5, age 9 and age 15	SEM with CFA	CBCL Construct housing insecurity CTSPC	Adolescent behavior Childhood housing insecurity Harsh parenting	Housing insecurity predicted: Adolescent anxious/depressive behaviors Rule-breaking, aggressive, and anxious/depressive behaviors via psychological aggression in parenting	Not all differential pathways or compounding impacts could be accounted for Dis not include a general population comparison group: not possible to determine whether the level of adolescent aggression differed from typically developmental stage.
Nuttall et al.^46^	USA	235	Kindergarten-aged children's families	Wave 1: average age 6.0 years; wave 2: 6.97 years; wave 3: 7.99 years	LCA	CBCL MSSTB CRPBI CBS OPS	Child INT and EXT Children's caregiving reactions to interparental conflict Children's representations of caregiver competence Parental psychological autonomy support versus restriction Prosocial behavior Interparental conflict	88% children reported positive caregiving responses in a task of interparental conflict scenarios 2 latent classes: 1) parentification= ↑ child caregiving responses, ↑ parental control and ↓ caregiving competence, 2) non-parentification= ↓ child caregiving, ↑ caregiving competence, and ↓ control Growth Mixture Model with adjustment trajectories: shifts in class solutions. Parentification = 30% of the sample. Parentification class (vs. non-parentification): ↑ initial EXT but ↓ more rapidly ↑ initial INT that ↑ more rapidly ↓ prosocial behavior, while non-parentification class showed ↑ over time Children in parentification class: resilience and normative functioning despite experiencing family parentification risk Familial pattern of parentification: mothers with poorer parenting than fathers	Children's actual caregiving responses not directly captured, potentially limiting validity Assessment of parentification did not clearly distinguish between emotional and instrumental forms Assessment did not differentiate whether caregiving reactions were directed towards the mother or the father, failing to capture potential differences in familial responses to parentification Two-parent nature of sample: may have influenced results Low parentification scores Small sample size
Rinne et al.^47^	USA	Study 1: 362 Study 2: 125	Study 1: women Study 2: mother-child pairs	0-5	LCA	EPDS CES-D CBQ NTCB	Trajectories of maternal depressive symptoms Child temperament Child executive function	Study 1 – Trajectories of maternal depressive symptoms: 4 trajectory classes: low-stable, persistent, increasing, and decreasing Changes in symptoms: linked to maternal ratings of child temperament and performance on executive function task Changes in symptoms: correlation with differences in child self-regulation Study 2 – Associations with offspring developmental outcomes in child follow-up: Children of women with ↓ symptoms: ↓ maternal ratings of effortful control at age 4 Children of women with ↑ symptoms: ↓ inhibitory control task at age 5 Preconception and postpartum depressive symptom: ↓ effortful control and inhibitory control outcomes, respectively	Small group sizes within some trajectories due to the majority of women exhibiting low-stable symptoms Participation bias Variability in time interval between the nearest preconception visit and pregnancy Differences among rating scales used for depressive symptoms: required standardization for comparison across measures Maternal depressive symptoms in first trimester of pregnancy as well as between postpartum and early childhood: not assessed
Bussemakers et al.^48^	UK	18,818	Children born 2000-2002	5-14	MLR	SDQ Age-appropriate vocabulary tests Family dysfunction variables Financial resources measures	Behavioral development Cognitive development	Children with household dysfunction: more behavioral problems already had ↓ verbal ability and ↑ behavioral problems prior to the onset of dysfunction Household dysfunction: ↓ parents’ financial resources ↑ odds of falling into poverty	Absence of unobserved factors Selection bias Limited measurements Limited generalizability Self-report Longitudinal measurement limitations Lack of detailed exploration
Barnhart et al.^49^	USA	5,510	Youth participants	9-10	PA SEM	CBCL YSR/BPM PALES MFES/FCS	Child INT and EXT ACEs Family conflicts	↓ family SES: predicted ↑ INT directly associated with ↑ ACEs ACEs: associated with↑ INT and↑ EXT Indirect effects between family SES, ACEs, and INT: stronger at ↑ levels of family conflict Family conflict moderated relation between family SES and ACEs, as well as association between family SES and INT	Secondary data limitation Family environment assessment: limited to negative attributes No control for ACEs or psychopathology at the beginning of the study. Limitations in family SES measure Discrepancies in the responses of parents and youths regarding symptoms and family conflicts

AAPI = Adult-Adolescent Parenting Inventory; ACE = adverse childhood experience; ALEQ = Adolescent Life Events Questionnaire; ALSPAC = Avon Longitudinal Study of Parents and Children; ANOVA = analysis of variance; AQS-3 = Attachment Q-Set version 3; ASA = Attachment Script Assessment; BDI = Beck Depression Inventory; BDIST = Battelle Developmental Inventory Screening Test; BPM = brief problem monitor; CBCL = Child Behavioral Checklist; CBQ = Children Behavior Questionnaire; CBS = Child Behavior Scale; CCSC = Children's Coping Strategies Checklist – modified version; CDI = Child Depression Inventory; CDS = Child Development Supplement; CES-D = Center of Epidemiological Studies-Depression Scale; CFA = confirmatory factor analysis; CFSS = Children's Food Security Scale; CIB = Coding Interactive Behavior; CIDI-SF = Composite International Diagnostic Interview Short Form; CPIC = Children's Perception of Interparental Conflict Scale; CPS = Conflict and Problem-Solving Scales; CRI = Conflict Resolution Inventory; CRPBI = Child Report of Parenting Behavior Inventory; CSI-4 = Child Symptom Inventory 4; CTSPC = Parent-Child Conflict Tactics Scale; DCI = Dyadic Coping Inventory; DESC = Divorce Event Schedule for Children; DFSCA = Drug Free Schools Outcome Study Questions; DPAS = Deviant Peer Affiliation Scale; EC = executive control; ECBQ = Early Child Behavior Questionnaire; EC-HOME = Early Childhood HOME Observation for Measurement of the Environment; EOI = emotional overinvolvement in parents; EPDS = Edinburgh Postnatal Depression Scale; EXT = externalizing problems; FCA = foundational cognitive abilities; FCS = Family Conflict Scale; FFCWS = Fragile Families and Child Well-being Study; FMSS = Five Minute Speech Sample; FPST = Family Problem-Solving Task; GHQ-28 = General Health Questionnaire-28; HOME = Home Observation for Measurement of the Environment; INT = internalizing problems; IPST = Interparental Problem-Solving Task; LCA = latent class analysis; LISRES = Life Stressors and Social Resources Inventory; LONGSCAN = Longitudinal Studies of Child Abuse and Neglect; LSAC = Longitudinal Study of Australian Children; LSDQ = Loneliness and Social Dissatisfaction Questionnaire; MANOVA = multivariate analysis of variance; MCDI = MacArthur Communicative Development Inventory; MCQ = Marital Communication Questionnaire; MFES = Moos Family Environment Scale; MLR = mMultiple logistic regression; MSSTB = MacArthur Story Stem Battery; NCE = neighborhood collective efficacy; NCP = neighborhood concentrated poverty; NEs-SEFQ-SF = Negative Expressive subscale of Self-Expressiveness in the Family Questionnaire, short form; NICHD SECCY = National Institute of Child Health and Human Development Study of Early Child Care and Youth Development; NLSAH = National Longitudinal Study of Adolescent Health; NLSCY = National Longitudinal Survey of Children and Youth; NRI = Network of Relationships Inventory; NSCAW-II = National Survey of Child and Adolescent Well-Being; NTCB = NIH Toolbox Cognition Battery; OPS = O’Leary-Porter Scale; PA = path analysis; PAES = Parental Alcohol Experiences Scale; PALES = PhenX Adverse Life Events Scale; PERI-D = Psychiatric Epidemiology Research Interview – Demoralization Scale; PRQ = Parenting Relationship Questionnaire; PSI = Parent Stress Index; RCMAS = Children's Manifest Anxiety Scale-Revised; RRPS = Rutter Revised Preschool Scales; SCID = System for Coding Interactions in Dyads; SCID-DSM-IV = Structured Clinical Interview for the Diagnostic and Statistical Manual of Mental Disorders, 4th edition (DSM-IV); SDQ = Strengths and Difficulties Questionnaire; SEM = structural equation modeling; SES = socioeconomic status; SILS = Shipley-Hartford Institute of Living Scale; SIMS-PR = Security in the Interparental Subsystems Scale; SSB-A = Story Stem Battery for Adolescents; SSRS = Social Skills Rating System; STAI = State-Trait Anxiety Inventory; STRS = Student-Teacher Relationship Scale; SWPS = Satisfaction With Parenting Scale; TOF = Test Observation Form; UCLA-LSIC = UCLA Life Stress Interview for Children; VBD-WPPSI-III = Vocabulary and Block Design sub-test of Wechsler Preschool and Primary Scale of Intelligence-III; VEX-R = Violence Exposure Scale for Children; WFCS = Work-Family Conflict Scale; WJ-III = Woodcock-Johnson-III; WPPSI-R = Wechsler Preschool and Primary Scale of Intelligence – Revised; YSR = Youth Self-Report Scale. ↑ = higher or increased; ↓ = lower or decreased.

## Discussion

We reviewed 43 articles (13 cross-sectional and 30 longitudinal) to assess which factors of stress and resilience in the family environment were associated with child development. Most of the studies were conducted in the United States. The most frequently used instruments to assess child general psychopathology were the CBCL and SDQ; and to evaluate child depressive symptoms, the CES-D and CDI. Multiple logistic regression and structural equation modeling were the most common statistical analyses used in cross-sectional and longitudinal studies. Associations between parental depression and youth internalizing symptoms and behavioral problems were reported in 16 (37.21%) studies.^1,2,6-8,18,27,29,30,32,36,38-40,45,48^ Along the same lines, positive parenting practices were positively associated with improved child development. A cohesive home environment was associated with low child stress and good socio-emotional development.^5,14,20,24,32,41,44^

Parental mental health problems, especially maternal depression, were not only associated with child mental health problems, but also with child developmental delays.^2,5,6,8,11,14,18,19,24,27,29,33-36,40,47,48^ Maternal postnatal depression affects children both through direct mother-infant interaction and also because of reduced caregiving for the child.^2^ In instances where maternal depressive symptoms were more pronounced, mothers who conveyed fewer primary control and secondary control coping messages were associated with children displaying elevated levels of internalizing symptoms during a task involving parental coping messages. This task was centered around the observation of discussion-based peer stress interactions.^19^ Rinne et al.^47^ demonstrated that maternal depressive symptoms presented four trajectory classes: low-stable, persistent, increasing, and decreasing. Offspring of mothers exhibiting escalating symptoms scored lower on an inhibitory control task at the age of five.

Mothers with major depression might present depressed and irritable mood, low concentration, and psychomotor alterations; therefore, these mothers could be hostile, pay little attention, and be slow to respond to their children.^35^ Observational studies of depressed mothers showed that such mothers had less affection and less interaction with their children.^29,35,36^ Moreover, unpredictable maternal mood and behavior seemed to be associated with risk for child mental health problems.^50^ Indeed, Vakrat et al.^36^ reported that maternal depression was associated with low parent sensitivity and poor child social engagement with parents, as well as less family cohesion.

In our review, we identified that parents with poor parenting abilities had children with more internalizing and externalizing symptoms, like worse communication, low emotional expression, insecurity, low social engagement, less organization and poorer hygiene, inattention, physical aggression, and poor developmental outcomes and well-being.^2,6-8,11,24,26,27,30,32,34,36,38,41,42,46,48^ Interventions focusing on parental mental health could lead to better child developmental outcomes. A Canadian population-based study found that identifying and supporting mothers who experience high anxiety symptoms in the perinatal period may mitigate the risk of developmental delays.^51^ A recent clinical trial showed the effectiveness for sustaining remission of maternal depression of an intervention that focused on sleep, routines, and self-care. This positive effect lasted for at least 2 years after childbirth.^52^ In the same direction, a scoping review about interventions offered to at-risk families, such as adolescent mothers and parents with severe mental health problems, in the 1st year of life concluded that they improved child behavior, parent-child relationship, and maternal sensitivity post-intervention. However, this review found no improvement in child cognitive development or internalizing or externalizing symptoms.^53^ According to a German clinical trial, multisystemic family therapy for child abuse and neglect alleviated parental psychological distress even 6 months after the intervention ended.^54^ This suggests that interventions can have a positive impact on child development.

A current theme linked to poor parenting abilities is "phubbing," the behavior of paying more attention to one's mobile device, particularly during social interactions, rather than giving full attention to the people present. Lv et al.^27^ report that maternal phubbing had a detrimental impact on emotional and behavioral issues in young children, highlighting a negative association between maternal phone use during interactions and the emotional well-being of the children. Phubbing disrupts mother-child bonds, which is a mediating factor in healthy child development, potentially leading to insecure attachments and subsequent behavioral problems in children. Also, daily hassles, minor and frequently occurring stressful life events, predicted change in the secure base script (i.e., the memory of essential childhood attachment experiences with the binding figure, used in new attachment relationships, e.g., caregiving and romantic relations).^37^

Positive parenting practices and positive parental feelings towards pregnancy have been positively associated with the child's increased resilience, communication, and social development.^14^ There is a dose-response relationship between positive parenting practices and the child's likelihood of experiencing developmental, social, or behavioral delays.^20^ Storytelling and singing to the child, for instance, were considered as positive parenting practices and associated with better child behavioral development, mainly if they occurred daily.^14,20^ This is in line with a study by Waters et al.^37^ demonstrating the importance of essential attachment experiences in childhood. Positive parental feelings included maternal engagement with appropriate child-centered activities, positive perceptions of the child, positive parenting style, active parental coping efforts, reflecting a strong emotional attachment to the child, family cohesion, and enjoyment of the parental role.^14,21,23,24,44,55^ The child's nonverbal communication was also a predictor of resilience, suggesting that the more a child communicates effectively in a nonverbal way, the more likely they were to attain normative behavioral development. Savage-McGlynn et al.^14^ hypothesized that more nonverbal communication is expressed following positive maternal feelings towards their children. Examples of non-verbal communication include pointing to an exciting object, waving unprompted at someone, extending arms to be picked up, shaking head "no," nodding head "yes," opening and closing hand to ask for an item, and blowing a kiss from a distance, which are not as often observed among children of depressed mothers. Researchers and healthcare professionals should share information about these positive parenting practices and their impact on a child's development to increase adherence to these practices. Furthermore, resilience among adolescents was associated with younger age, better parent-child relation quality, and neighborhood safety, while physical abuse history and affiliation with deviant peers decreased the odds of adolescents remaining resilient.^44^

Also, poorer development delays may be significantly associated with a vulnerable social environment, such as poverty level, immigrant mother, and parents’ educational status, as referenced by studies carried out in developed countries.^20,22^ Housing insecurity predicted anxious and depressive behaviors and decreased verbal ability in adolescents.^45,48^ It is well established in the literature that improving neighborhood conditions^56,57^ and socioeconomic status of the population,^58,59^ as well as incentivizing strategies to increase social integration and cohesion^60,61^ can improve mental health outcomes, particularly among children.

Findings from our scoping review showed that a cohesive home environment was associated with low child stress and good socioemotional development.^5,23,32,35^ Family environments with chronic strain and stress,^33^ mainly between parents, were associated with worse relationships and future depressive symptoms among children.^16^ Marital problems (e.g., divorce or conflicts or non-supportive co-parenting) were also associated with low child development, internalizing symptoms, behavioral problems, and harsh parenting.^5,15,29,41,42,45,48^ Moreover, using the Five-Minute Speech Simple (FMSS) scale, which evaluates the emotional climate of the parent-child relationship in the caregiver's speech, parental self-sacrifice and overprotection were associated with boys’ externalizing symptoms.^38^ In fact, all these factors seem to play a role in children's outcomes. Maternal parenting stress and exposure to adverse childhood experiences serve as mediators between neighborhood attributes and outcomes among adolescents. Parenting stress played a mediating role in the connections between neighborhood poverty and collective efficacy and outcomes in adolescents. Maternal parenting stress, exposure to adverse childhood experiences, neighborhood collective efficacy, and mothers’ cohabitation status were predictors of adolescents’ behavior problems.^39^ All these studies show that a cohesive family creates a more supportive environment, which decreases the chances of socioemotional developmental delays in the children.

Another marker of the family is their caregiver characteristics. Having an immigrant mother with low educational attainment was associated with delays in child development in Canada.^22^ Likewise, delayed development was also observed among children raised only by their grandparents in one study in Thailand, that has not been evaluated for quality.^21^ A trial that evaluated therapy interventions for grandparents raising grandchildren found they improved grandmothers’ psychological distress and emotional expressions and led to better development of parenting practices and therefore reduced internalizing and externalizing symptoms among their grandchildren aged between 4 and 12.^62^

Healthcare professionals and teachers could be essential catalysts to promote the healthy socioemotional development of children.^41,43,54^ These professionals must establish a solid parent-professional partnership with family-centered strategies. This bond may enable families to request these professionals’ support when needed, and the professionals can assist families who are dealing with developmental issues, such as difficulties in social interaction or emotional communication.^63^ Given that interventions targeting parental mental health could lead to better child developmental outcomes, early identification of such issues should focus on public health, as early child care has been demonstrated to positively impact children's cognition, socioemotional development, and behavior.^64^

Despite the insights gained from the literature review, some limitations warrant discussion. One limitation was the inclusion of only publications written in English or Portuguese. Although this is a limitation, previous research shows that language restrictions do not alter the main findings of systematic reviews.^65,66^ Another limitation was that our search strategy may have lacked sensitivity, particularly by omitting significant search terms such as "mother" or "father," but we checked all references in the selected articles and we contacted experts in the field who suggested additional articles. Further limitations were the heterogeneity and lack of standardization of the articles reviewed regarding the statistical analyses used in each study, the small sample sizes of the majority of articles, and the fact that the definitions of "resilience" and "family cohesion" varied considerably among the studies. Another limitation was the broad spectrum of our search. Although this entails that our study takes a less specific look at the issue, we aimed to privilege the bigger picture of the current scenario of literature on this topic, raising a more comprehensive discussion of potential gaps identified. Finally, an additional limitation was not using scales to evaluate bias or methodological quality in the articles included.

In conclusion, this study suggests that the family environment is an essential determinant of child socioemotional development. This review's strength is that we produced a synthesis of the main findings on the impact of stress and resilience on child development and on the relationship between parental mental health problems, particularly maternal depression, and youth internalization and externalization problems, as well as showing the significant importance of positive parenting practices in promoting healthier child development. Moreover, a cohesive home environment was associated with low child stress and good socioemotional development. Vulnerable social environments, such as poverty level and housing insecurity may be linked to child mental health problems. Promoting a better family dynamic and improving family cohesion and parenting abilities could benefit the child's socioemotional development. Also, increasing family and child resilience increases quality of life within family units. Therefore, this is an entire area of study and intervention for mental health professionals. Future studies are needed to identify public policy models that address the family environment and promote social and health improvements. Future research could also shed light on the factors underlying families’ resilience and stress starting in early childhood.
